# Quantitative Analysis and Health Risk Assessment of Heterocyclic Aromatic Amines in Plant-Based Milk Beverages

**DOI:** 10.3390/foods14193295

**Published:** 2025-09-23

**Authors:** Alejandro Mandelli, Adriana Bochetto, María Guiñez, Soledad Cerutti

**Affiliations:** 1 Instituto de Química de San Luis (Consejo Nacional de Investigaciones Científicas y Técnicas), Laboratorio de Espectrometría de Masas, Área de Química Analítica, Facultad de Química Bioquímica y Farmacia, Universidad Nacional de San Luis, Bloque III, Ejército de los Andes 950, San Luis CP 5700, Argentina; alemandelli87@gmail.com; 2 Facultad de Ingeniería y Ciencias Agropecuarias, Universidad Nacional de San Luis, Campus Universitario, Ruta Prov. N° 55 (Ex 148) Extremo Norte, Villa Mercedes, San Luis CP 5730, Argentina; abochetto@gmail.com

**Keywords:** heterocyclic aromatic amines, plant-based milk, µSPE, thermal processing, cancer risk assessment

## Abstract

Heterocyclic aromatic amines (HAAs) are mutagenic and potentially carcinogenic compounds formed during the thermal processing of protein- and sugar-rich foods, yet their occurrence in plant-based milk alternatives remains largely unexplored. To the best of our knowledge, this is the first study to report on the presence of HAAs in plant-based milk beverages. The aim of this study was to develop a robust and environmentally friendly µSPE–UHPLC–MS/MS method for the quantification of ten HAAs in plant-based milk beverages and to assess the potential health risks associated with their formation under varying thermal treatment conditions. A novel analytical method was applied to both commercially available and homemade beverages prepared from almonds, soy, cashews, and peanuts, including pasteurized and unpasteurized variants with and without added sugar. Chemometric tools were used to optimize retention and enrichment strategies. Detection limits ranged from 0.01 to 0.04 µg L^−1^, while quantification limits ranged from 0.01 to 0.05 µg L^−1^. Recovery rates ranged between 84% and 100%, with enrichment factors spanning 43 to 50. HAA concentrations varied from 0.09 to 13.66 µg L^−1^, with significantly higher levels observed in beverages subjected to thermal treatment, particularly those with added sugar and higher protein content. The health risk assessment indicated that the cumulative incremental lifetime cancer risk (ILCR) values were below the unacceptable threshold (10^−4^), though some scenarios approached 10^−5^, suggesting a moderate risk for frequent consumers of plant-based milk alternatives.

## 1. Introduction

The rising awareness of health and environmental issues is rapidly increasing, resulting in a daily substitution of animal products—which are linked to a higher risk of diseases, cancer, and diabetes—with plant-based options. A significant result of this dietary change is the growing popularity of plant-based milk alternatives (PBMAs) [1].

Plant-based milk, also known as extracts of plants, offers essential nutrients necessary for growth and development. These alternatives are particularly rich in vitamin B, dietary fiber—which may support digestion—variable protein content, and healthy fats while being free from lactose, making them suitable for individuals with lactose intolerance, and cholesterol—which is important for cardiovascular risk reduction The FDA (U.S. Food and Drug Administration) defines plant-based products marketed and sold as milk alternatives as those made from nuts (such as hazelnuts, walnuts, cashews, almonds, etc.), seeds (like sesame and flax), rice, oats, or legumes such as soy [2,3,4]. The food production chain, along with anthropogenic activities, significantly contributes to the presence or formation of persistent organic pollutants in food. Several phases of this process can lead to detrimental impacts on food quality, which may include nutrient depletion, the creation of toxic compounds, or reaction intermediates that get integrated into the food structure [5,6,7,8]. There has been a notable interest in researching, managing, and tracking pollutants that could pose health risks, especially those known as emerging pollutants. One category of these compounds, known as heterocyclic aromatic amines (HAAs), consists of complex organic molecules that are characterized by their multi-ring aromatic structures and are classified into two main categories [9,10] (listed in Table S1). Thus, the thermal group includes compounds formed from reactions involving free amino acids, creatine, and hexoses at temperatures between 150 and 300 °C, a process known as a Maillard reaction. This reaction leads to the formation of heterocyclic pyridines and pyrazines, which can further transform into imidazoquinoxalines. Examples comprise the following: 2-amino-3,4,8-trimethyl-imidazo [4,5-f]-quinoxaline (4,8-DiMeIQx); 2-amino-1,6-dimethylimidazo[4,5-b]pyridine (DMIP); 2-amino-3-methyl-imidazo[4,5-f]-quinoline (IQ); 2-amino-3,4-dimethyl-imidazo[4,5-f]-quinoline (MeIQ); 2-amino-3,8-dimethylimidazo[4,5-f]-quinoxaline (MeIQx); and 2-amino-1-methyl-6-phenylimidazo[4,5-b]pyridine (PhIP). Another group, known as the pyrolysis group, is formed because of the pyrolysis of amino acids and proteins at temperatures above 300 °C. Within this group, the following compounds can be listed: 2-amino-9H-pyrido[2,3-b]indole (AαC), 2-amino-3-methyl-9H-pyrido[2,3-b]indole (Me AαC), 3-amino-1,4-dimethyl-5H-pyrido[4,3-b]indole (Trp-P-1), and 3-amino-1-methyl-5H-pyrido[4,3-b]indole (Trp-P-2) [11,12]. The International Agency for Research on Cancer (IARC) has classified specific HAAs as possible and probable human carcinogens, assigning them to Class 2B and 2A. These compounds are over 100 times more mutagenic than aflatoxin B1 and over 2000 times more so than benzo[a]pyrene. Furthermore, the European Commission advises that the daily intake of HAAs should not exceed 1 μg per person each day [13,14].

Pasteurization is a technological process designed to extend product shelf life, classified into three categories based on temperature and duration: Low-Temperature Long-Time (LTLT), High-Temperature Short-Time (HTST), and Ultra-High Temperature (UHT). The LTLT method requires a temperature of no less than 62.8 °C for a minimum duration of 30 min. In contrast, the HTST method requires at least 71.1 °C for 15 s. The UHT method operates at a minimum temperature of 135 °C for just 1 s. UHT milk can be stored for several months without refrigeration, whereas pasteurized milk, when kept at temperatures below 6.1 °C, has a shelf life ranging from 10 to 20 days [15,16,17]. However, heat treatment may induce undesirable alterations in milk, including the formation of Maillard reaction products and other thermally derived compounds such as heterocyclic aromatic amines, which are predominantly produced during the processing of protein-rich foods [18]. A Maillard reaction is a non-enzymatic browning process initiated by the interaction of amino groups from amino acids or proteins with carbonyl groups of reducing sugars, resulting in the generation of aroma-active compounds and brown melanoidins that contribute to the characteristic flavor, color, and texture of heated foods. The reaction rate is strongly influenced by processing parameters such as temperature, pH, reaction time, and water activity. Although this reaction enhances desirable sensory attributes, it may also lead to the formation of unwanted compounds with potential health implications. In contrast, enzymatic browning involves the oxidation of phenolic compounds catalyzed by polyphenol oxidase in the presence of oxygen, producing brown pigments without the requirement for heat [18].

To accurately quantify these harmful contaminants in food products, it is essential to use analytical methods that are not only sensitive and selective, but also environmentally friendly. These methodologies should include extraction, cleanup, and enrichment processes to reduce interferences from complex food matrices and to enhance the analytical signal, which is crucial for a successful trace analysis [19].

The most used protocols for the extraction of these contaminants include supercritical fluid extraction, microwave-assisted extraction, Quick, Easy, Cheap, Effective, Rugged, and Safe (QuEChERS) extraction, liquid–liquid microextraction (µLLME), and solid-phase microextraction (µSPME). Among these methods, µSPME is preferred owing to its high sensitivity and reproducibility as well as its separation capabilities [20].

The comparative analysis of extraction methodologies (as summarized in Table S2) highlights both the analytical robustness and sustainability aspects of the approach developed in this study.

Separation and detection methods for HAAs include techniques such as high-performance liquid chromatography (HPLC) combined with fluorescence detection, UV absorbance, or mass spectrometry (MS). The use of MS is particularly advantageous for detecting HAAs due to their structural properties, which produce characteristic intense fragments. When interfaced with liquid chromatography, these techniques enable highly efficient detection of carcinogens at low concentrations in food samples, ensuring both selectivity and sensitivity in the analytical assessments. According to reports, HAAs have been found in a variety of matrices, including meat, roasted coffee, alcoholic beverages, cheese, toasted bread, eggs, and the breast milk of healthy mothers. Nevertheless, few studies have reported the occurrence of these contaminants in milk from animals, and even less attention has been given to their occurrence in dairy alternatives like plant-based beverages, which are becoming more popular among consumers [16,21,22].

This study represents the first comprehensive study reporting the occurrence of heterocyclic aromatic amines in plant-based milk alternatives, offering new insights that connect formulation and processing factors with HAA formation and the associated health risks. Therefore, this research aimed to develop and apply a µSPME-UHPLC-MS/MS method based on a poly(MAA-co-EDMA) monolithic sorbent for the determination of heterocyclic aromatic amines in plant-based milk alternatives. The synthesis, characterization, and analytical performance of the sorbent for the extraction of HAAs have been comprehensively detailed in a prior publication by our research group [20]. The proposed methodology was employed to quantify ten HAAs: 4,8-DiMeIQx, DMIP, IQ, MeIQ, MeIQx, PhIP, AαC, Me AαC, Trp-P-1, and Trp-P-2 in plant-based milk alternatives The methodological parameters were optimized through chemometric tools, specifically the Statistical Design of Experiments and Artificial Neural Networks (ANN), aimed at reducing the number of experiments conducted while maximizing the acquisition of information in a short period. The experimental conditions were applied to samples of plant-based beverages, specifically including almond, soy, cashew, and peanut varieties. The methodology successfully determined the heterocyclic aromatic amines in 31 samples from both commercial and homemade sources. In the homemade category, factors such as the addition of sugar, pasteurization, and their combined effects on HAA formation were considered. Additionally, the ecological dimensions of the proposed methodology were evaluated using the green metric AGREEprep and the Blue Applicability Grade Index (BAGI) [23,24]. Furthermore, the potential impacts on human health were evaluated through an incremental lifetime cancer risk analysis (ILCR) associated with the consumption of HAAs in plant-based milk alternatives [25,26].

## 2. Materials and Methods

### 2.1. Chemicals and Reagents

The heterocyclic aromatic amines evaluated in this study, namely 4,8-DiMeIQx, DMIP, IQ, MeIQ, MeIQx, PhIP, AαC, MeAαC, Trp-P-1, and Trp-P-2, were obtained from Toronto Research Chemicals Inc. (North York, ON, Canada) and have a purity greater than 98%. Optima^®^ LC-MS grade acetonitrile (ACN) and water were purchased from Fisher Scientific (Fair Lawn, NJ, USA). Formic acid (FA) was obtained from Fisher Scientific (Fair Lawn, NJ, USA). Ultrapure water, with a resistivity of 18.2 MΩ cm^−1^, was produced using a Milli-Q water purification system manufactured by EASYpure (RF Barnstead, Dudubuque, IA, USA). The reagents used for the synthesis of the poly(MAA-co-EDMA) monolithic material included methacrylic acid, ethylene glycol dimethacrylate (EDMA), and 2,2′-azobisisobutyronitrile (AIBN), all obtained from Sigma-Aldrich (St. Louis, MO, USA).

Standards were prepared daily in a solvent mixture of water and acetonitrile (30:70, *v*/*v*) through serial dilution of 10 mg L^−1^ stock solutions of each HAA and were stored at −18 °C until needed. Calibration curves for both the external (pure solvent) and sample-spiked conditions were generated by preparing individual and mixed solutions at various concentration levels ranging from 0.01 µg L^−1^ to 100 µg L^−1^. The solutions were stored at 4 °C, protected from light, and contained in amber flasks.

The laboratory equipment included a blender (Philips Daily Collection, 550W, mode LHR2125), an ultrasonic water bath (Test lab TB-10TA, Buenos Aires, Argentina), a vortex (ArcanoHX-2000-1, Buenos Aires, Argentina), an electronic microbalance with a readability of 0.1 mg, specifically the UMX2 model (Ohaus, Nänikon, Switzerland), a centrifuge (U-320R-BOECO, Hamburg Germany), and a 20-port extraction manifold (Waters, Milford, CT, USA) paired with a High-Capacity Vacuum Pump (Gast DOA-P504-BN, IDEX, LV, USA).

### 2.2. Synthesis of Organic Poly(MAA-co-EDMA) Monolithic Material and Assembly of the µSPE System

In the µSPE stage, the synthesis of novel materials for solid-phase extraction was carried out under conditions like those outlined in a previous study by our group [20]. The procedure involved preparing a polymer mixture of methacrylic acid, ethylene glycol dimethacrylate, acetonitrile, and 2,2′-azobisisobutyronitrile, which was sonicated, degassed, and polymerized in an oven. The resulting material was crushed, sieved, and packed into a solid-phase extraction (SPE) cartridge, with frits placed at both ends and 1 mL of water used to distribute the material evenly before extraction.

### 2.3. Sample Collection and Preparation

A total of 31 samples of plant-based milk were analyzed, which included 15 commercially produced samples and 16 homemade samples. All commercial plant-based samples were purchased from local supermarkets in San Luis, Argentina, and included the following varieties: almond (*n* = 12), soy (*n* = 1), cashew (*n* = 1), and peanut (*n* = 1). The homemade samples were prepared using almonds (*n* = 4), soy (*n* = 4), cashews (*n* = 4), and peanuts (*n* = 4). These homemade samples were formulated based on the commercial beverages, following specific ingredient proportions and incorporating the following variations: (i) Homemade sample, unsweetened and unpasteurized (H); (ii) Homemade sample, sweetened and unpasteurized (HS); (iii) Homemade sample, unsweetened and pasteurized (HP); (iv) Homemade sample, sweetened and pasteurized (HSP); and (v) Commercial sample, sweetened and pasteurized (CSP). A brief overview of the preparation methods for each type of plant-based beverage is provided below.

For the almond-based beverage, the almonds were soaked in water for 12 h at a temperature of 4 °C. After soaking, the dried fruits were ground and mixed with distilled water in a 1:5 (*w*/*v*) ratio for 2 min. The mixture was then filtered and set aside for later use [27,28].

For the preparation of soy milk, soybeans were soaked in distilled water for 12 h at 4 °C. The soaked soybeans were then boiled in fresh, sterile distilled water for 30 min. After boiling, the soybeans were ground with water at room temperature in a 1:5 (*w*/*w*) ratio. The resulting mixture was heated to 60 °C and filtered to produce soy milk, which was stored for future use [27].

The production of cashew-based milk involved the following steps: Cashews were soaked in distilled water for 6 h at 4 °C. Next, the mixture was blended while maintaining a 1:3 (*w*/*v*) ratio of cashews-to-water until a total volume of one liter of product was obtained. The resultant extract was filtered and stored at 4 °C until use [29].

Peanut-based milk was made by mixing dried peanut grains in a 1:9 (*w*/*v*) ratio. The grains were soaked for 12 h at 4 °C before being blended in a mixer for 2 min. After blending, the mixture was filtered and kept in the refrigerator until needed [30].

An amount of 30 g of sugar per liter was added to sweetened beverages, as indicated by different brands for each type of nut, seed, or cereal, while considering the nutritional labels of commercial beverages [29,31]. After processing each type of beverage, the final volume was split into four portions, as previously outlined. For the plant-based beverages that were pasteurized, the process employed was low-temperature long-time (LTLT) pasteurization. This method involves heating the beverages to a minimum temperature of 63 ± 1 °C for no less than 30 min.

The total volume of the samples to be studied was split into three replicates, which included samples that were not pasteurized or sweetened, unpasteurized samples with added sugar, pasteurized samples without sugar, and pasteurized samples with sugar.

### 2.4. Analytes Extraction, Cleanup, and Enrichment

Plant-based milk alternatives are emulsions made up of various components, including carbohydrates, proteins, fats, minerals, and, sometimes, additives and stabilizers. This complex composition makes it difficult to identify trace contaminants. Furthermore, amines have the potential to interact non-specifically with proteins, which necessitates that the precipitating agent effectively dissociates these proteins from the sample matrix. Consequently, it is essential to precipitate and separate proteins, as well as to suspend the components, before determining HAAs. To facilitate precipitation, several researchers have employed aqueous acid solutions, like trichloroacetic or perchloric acid, along with solvents such as acetonitrile or methanol [32]. Before the SPE procedure, the sample was prepared as follows: A 20 mL aliquot of PBMAs was placed in a thermostatic bath until it reached a temperature of 30 °C. Then, a 2.5 mL solvent mixture of water and acetonitrile (30:70, *v*/*v*) with 3 % formic acid was added. The mixture was shaken continuously for 30 s and then centrifuged for 10 min at 4000 rpm at 4 °C. Finally, the supernatant was filtered, and 10 mL of the sample was collected and introduced into the µSPE system. The solid-phase extraction procedure was as follows: (i) the system was conditioned before use with 1 mL of methanol (MeOH), followed by 1 mL of water (H_2_O); (ii) a sample volume of 10 mL was loaded at a rate of 2 mL min^−1^; (iii) the system was then rinsed with 1 mL of water; and (iv) analytes were eluted using a solvent mixture of water and acetonitrile (30:70, *v*/*v*) with 0.1 % formic acid at a flow rate of 1.75 mL min^−1^. The resulting eluate was filtered through a nylon syringe filter with a pore size of 0.22 µm and placed into amber vials for UHPLC-MS/MS analysis. The cartridges were reconditioned by repeating the second step to maintain their reusability. A schematic representation of the procedure is represented in Figure S1.

### 2.5. Instrumental Analysis

The chromatographic conditions and mass spectrometric instrumental parameters were consistent with those reported in previous works from our group [11,33]. The equipment used for the ultra-high-performance liquid chromatography analyses was an Acquity™ Ultra High-Performance LC system (Waters, Milford, CT, USA). Chromatographic separation was performed by injecting a sample volume of 10 μL into an ACQUITY UPLC^®^BEH Shield RP 18 column (Waters, Milford, CT, USA), with a 2.1 mm internal diameter × a 100 mm length and a 1.7 μm particle size. The binary mobile phases consisted of water (A) and acetonitrile (B), each containing 0.1% formic acid. The mobile phase was delivered at a flow rate of 0.25 mL min^−1^, with a total running time of 5 min, and the column temperature was kept at 30 °C. The selected chromatographic conditions resulted in good peak shapes within the brief analysis period.

Mass spectrometric analysis was performed on a Quattro Premier™ XE Micromass MS Technologies instrument, which features a triple quadrupole analyzer and is equipped with an ESI interface. The ionization source was operated in a positive mode for each HAA, and data were acquired using the multiple reaction monitoring mode (MRM) of selected ions at the first (Q1) and third quadrupoles (Q3). To select fragment ions of m/z (Q1) → m/z (Q3), a solution of the standard in acetonitrile was directly injected into the spectrometer using an injection pump. The data obtained were processed using MassLynx Mass Spectrometry Software, version 4.1 (Waters, Milford, CT, USA).

### 2.6. Multivariate Optimization

HAAs are found in real samples at very low concentrations, typically just a few parts per billion. To address the challenges posed by complex food matrices, effective methods for extraction, purification, and enrichment are crucial. This research examined the factors affecting the extraction process, the elution conditions, the physicochemical affinity of the analytes, and strategies for miniaturizing solid-phase extraction. A two-level full factorial design (2^3^) was employed to identify the variables influencing retention and elution conditions. The significance of each variable was assessed through Pareto plots. In the evaluation of retention conditions, the variables were defined as follows: (A) mass of the adsorbent phase (mg); (B) volume of the sample being loaded (mL); and (C) sample loading flow rate (mL min^−1^). Conversely, to optimize elution conditions, the variables were defined as follows: (A) elution solvent volume (mL); (B) elution flow rate (mL min^−1^); and (C) composition of the eluent (expressed as the percentage of acetonitrile (ACN) in a mixture of water and acetonitrile (*v*/*v*)). Statistical analysis was performed using StatEase Design Expert. Further optimization was achieved through Artificial Neural Networks (ANNs). The factors evaluated included (A) the mass of the adsorbent phase (mg); (B) elution solvent volume (mL); (C) elution flow rate (mL min^−1^); and (D) the composition of the eluent (expressed as the percentage of acetonitrile (ACN) in a mixture of water and acetonitrile (*v*/*v*)), with the assistance of MATLAB Version 9.13.0 (R2022b) Update 2.

### 2.7. Validation of Analytical Methodology

#### 2.7.1. Analytical Sensitivity

The limit of detection (LOD) and the limit of quantification (LOQ) were established as the minimum detectable amounts of analyte, corresponding to signal-to-noise ratios of 3.3 and 10, respectively, as defined by the International Union of Pure and Applied Chemistry (IUPAC) [34,35].

#### 2.7.2. Working Range

The working or linear range (LR) of the calibration curves for the spiked PBMA samples was established through the least-squares linear regression analysis, which correlates the signal intensity to HAA concentrations. The linearity of the fitted model was assessed using an F-test within the working concentration range of 0.005 to 100 µg L^−1^. In this context, samples were prepared at five concentration levels, with each level analyzed in triplicate (*n* = 3).

#### 2.7.3. Recovery and Enrichment Factor

The importance of the variables that influence the analytical process is crucial for understanding its capabilities. For this reason, a defined volume of each sample (10 mL) was homogenized and spiked. The variables of the developed methodology have been optimized through the evaluation and comparison of the Percentage of Recovery (*R* (%)) and the Enrichment Factor (EF) [26,34].

(1) $$EF=\frac{Concentration\;of\;the\;analytes\;in\;the\;final\;extract}{Concentration\;of\;the\;analytes\;in\;the\;initial\;sample}$$

(2) $$R\left(\%\right)=\left[\frac{\left(C_{found}-C_{real}\right)}{\left(C_{added}\right)}\right]*100$$

where *C_found_* refers to the analyte concentration after adding a known amount of standard to the real sample, *C_real_* represents the analyte concentration in the real sample as well as *C_added_* denotes the known amount of standard that was spiked to the real sample.

#### 2.7.4. Precision

Method precision was assessed in terms of repeatability (intra-day precision) and reproducibility (inter-day precision). Reproducibility was tested using the same procedure across five separate days. Spiked samples, including 3 blanks and 3 replicates, at concentrations ranging from 0.05 to 2.5 µg L^−1^ were analyzed under the aforementioned conditions.

#### 2.7.5. Matrix Effect

The matrix effect (*ME* (%)) in both qualitative and quantitative analyses refers to the influence of sample components that may co-elute with the target analytes during chromatographic separation. These components may ionize simultaneously or interfere with the ionization of the target chemical species, leading to either suppression or enhancement of the analytical signal during detection [32]. To assess the effect of the sample matrix, the signal from spiked samples is compared to that from a spiked pure solvent, which is a mixture of water and acetonitrile (30:70, *v*/*v*) with 0.1% formic acid. The matrix effect was calculated as follows:

(3) $$ME\;(\%)=[100-\left(\frac{Slope\;(spiked\;sample)}{Slope\;(calibration\;solutions)}\right)\times 100]$$

#### 2.7.6. Carcinogenic Risk

Assessing the cancer risk associated with dietary exposure to HAAs is difficult due to the lack of validated Cancer Slope Factor (CSF) data from animal studies; however, the available CSF values, although limited and sometimes derived from extrapolations, represent the most authoritative references and are widely adopted in regulatory risk assessments, ensuring transparency and consistency in interpreting the results. CSF values quantify the probability of developing cancer as a consequence of exposure to specific substances. Given the significant carcinogenic potential of each HAA compared to other pollutants, which are classified as Group 2A or 2B carcinogens, an established oral slope factor has been used for the HAAs under study. The risk assessment for the intake of HAAs is based on the estimation of the quantity of these compounds consumed with a serving of plant-based milk alternatives, with a typical serving size of PBMAs being 200 mL. The estimated daily intake (*EDI*) is calculated as shown in Equation (6), whereas Equation (7) illustrates the chronic daily intake (*CDI*) [25,26].

(4) $$EDI=Mean\;consumption\left(\frac{L}{day}\right)\times C\;\left(\frac{mg}{L}\right)$$

(5) $$CDI=\frac{C\times IR\times ED\times EF}{BW\times AT}$$

*C* represents the concentration of HAAs expressed mg L^−1^; *IR*, denotes the recommended daily intake of plant-based milk, which is 2–3 servings of 200–250 mL each [25]; *BW* represents body weight in kilograms for children, adolescents and adults at 21, 49, and 65 kg, respectively; *ED* signifies the duration of exposure in years, which is 5, 13, and 84 for children, adolescents, and adults. *EF* indicates the frequency of exposure, set at 365 for all, and *AT* is the average time calculated as ED multiplied by 365 (days).

The Incremental Lifetime Cancer Risk (*ILCR*) quantifies the increased likelihood of cancer development that an individual may face over their lifetime. The term is defined as a reasonable upper-limit estimate of the probability that an individual may develop cancer at some point in their lifetime due to exposure to a contaminant, as illustrated in Equation (8). According to the United States Environmental Protection Agency, an ILCR value of less than 10^−6^ is regarded as negligible, while an ILCR value exceeding 10^−4^ is deemed unacceptable. Values between 10^−6^ and 10^−4^ require additional evaluation. In this work, it is postulated that there is an additive carcinogenic effect from HAA co-exposure. A cancer risk assessment was performed by calculating the cumulative ILCR linked to each HAA [25,26]:

(6) $$ILCR\;Total=\sum \left(CDI\times CSF\times ADAF\right)$$

In Equation (6), Chronic Daily Intake (*CDI*) refers to the calculated intake of heterocyclic aromatic amines (HAAs) expressed in milligrams per kilogram of body weight per day (mg kg^−1^ BW day^−1^) for both males and females across various age categories. The Cancer Slope Factor (*CSF*) represents the cancer slope factor, defined as the risk associated with a lifetime average exposure to one milligram per kilogram per day (mg kg^−1^ day^−1^) of a carcinogenic chemical; this factor is specific to each contaminant. Age-Dependent Adjustment Factors (*ADAF*) are used to account for variations in susceptibility based on age, with a value of 3 assigned to children aged 3 to 15 years, and a value of 1 applied to youth, adults, and senior populations.

This research calculated the total incremental lifetime cancer risk (*ILCR* Total) associated with heterocyclic amines, which include IQ, MeIQ, MeIQx, AαC, Me AαC, Trp-P-1, and Trp-P-2, based on previously established guidelines [25,26]. The CSF reference values for these compounds are 1.4, 1.5, 1.7, 0.4, 1.2, 2.6, and 3.2 (mg kg^−1^ day^−1^), respectively [36].

#### 2.7.7. Sustainability Assessment

The evaluation of the greenness of the method was conducted using AGREEprep (Analytical Greenness Metric for Sample Preparation), a metric tool designed to assess the greenness of the sample preparation stage within an analytical procedure. This tool is supported by open-source, open-access software that facilitates assessment by guiding the users through ten criteria and enabling the easy generation of results, either as a graph or in the form of a comprehensive report, as detailed in reference [23]. Furthermore, the Blue Applicability Grade Index (BAGI) is introduced as a novel metric for evaluating the practicality of an analytical method. This software is intended to enhance the functionality of existing green metrics-based tools, such as AGREEprep, with a primary emphasis on the practical aspects of White Analytical Chemistry. The tool evaluates ten key attributes, including the type of analysis, the number of analyzers that can be simultaneously determined, the number of samples that can be analyzed per hour, the types of reagents and materials used in the analytical method, the required instrumentation, the number of samples that can be treated simultaneously, the need for preconcentration, the degree of automation, the type of sample preparation, and the amount of sample, as described by Manousi et al. [24]. All the necessary data were input into both software (which are free to use). The total score is displayed at the center of each pictogram generated for AGREEprep and BAGI. In the first metric, the score is represented in a pictogram that ranges from red (0) to dark green (1). A score that is nearer to green or a value of 1 means a more sustainable process. In the second metric, a score nearing 100 indicates the higher viability and practicality of the analytical method developed.

## 3. Results and Discussion

### 3.1. Optimization of Retention and Elution Conditions

PBMAs are complex matrices where the precipitation and separation of proteins, fats, dissolved solids, and sugars play a crucial role. In addition, the development of a UHPLC-MS/MS method for quantifying pollutants with diverse physicochemical properties is quite challenging. As a result, a successful strategy includes optimizing a μSPE-based analytical technique to address a wide range of interactions. This study analyzed various factors such as the mass of the adsorbent material, the type of elution solvent, and the elution time to determine the best conditions for retention and elution. Experimental designs were employed to screen each variable, and the optimal conditions were assessed based on the extraction recovery efficiency (*R* (%)).

Initially, different volumes of solvents or mixtures were tested, including acetonitrile and methanol (from 1 to 10 mL), along with various combinations of these solvents and water in ratios of 25% to 75%. Some mixtures also contained formic acid at concentrations between 0% and 10% to improve precipitation. For subsequent experiments, a mixture of water and acetonitrile (30:70, *v*/*v*) with 3% formic acid was used, which demonstrated improved extraction efficiency for all the HAAs analyzed. This mixture facilitated effective phase separation during sample processing and resulted in minimal to no matrix effects on the signals obtained for each target compound.

Additionally, the following factors affecting analyte retention efficiency were optimized: (A) the mass of the adsorbent (mg); (B) the sample loading volume (mL); and (C) the sample loading flow rate (mL min^−1^). For variable (A), a significant increase in retention values was observed with the increase of the mass of the adsorbent material. However, no differences were found with masses greater than 10 mg of the poly(MAA-co-EDMA) monolithic material. Thus, the optimal conditions for this variable were examined at 5, 10, and 15 mg. Concerning sample loading volume (B), it is generally recognized that a larger quantity of analytes correlates with a greater mass or volume of the sample. Therefore, these conditions were optimized based on the volume of vegetable milk used for the extraction of HAAs, with retention percentage values (*R*%) calculated for sample volumes of 10, 25, and 40 mL. In this preliminary screening, the flow rate (C) at which the target substances pass through the µSPE system is of critical importance. This parameter facilitates sufficient interaction times and ensures that all retention sites on the material are fully occupied. Consequently, the sample loading flow rate was evaluated as a significant variable for analytical retention. For the purposes of this study, flow rates of 0.50, 1.25, and 2.00 mL min^−1^ were considered.

The parameters that influence the effective elution of the compounds were subsequently optimized, leading to a quantitative elution of 100% of the HAAs that were initially retained. The optimized parameters included (A) eluent volume, evaluated at 0.10, 0.25, and 0.40 mL; (B) eluent flow rate (mL min^−1^), studied at 0.50, 1.25, and 2.00 mL min^−1^; and (C) eluent composition, expressed as the percentage of acetonitrile (ACN) in the H_2_O/ACN (*v*/*v*) mixture, evaluated at 60%, 80%, and 100%.

**Figure 1:**
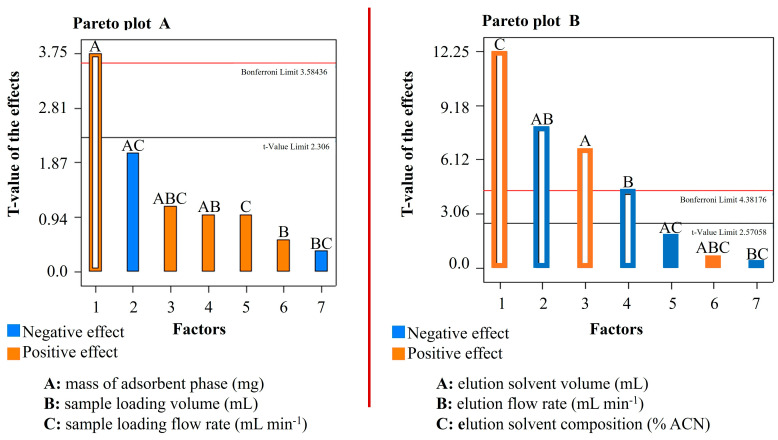
Pareto plots illustrating the effects of experimental factors on the analytical responses in the two μSPE factorial designs. (**A**) Retention conditions. (**B**) Elution conditions.

The primary significant effects on the analytical response for the two factorial designs applied are depicted in the Pareto plots: Figure 1A (retention conditions) and Figure 1B (elution conditions).

The aforementioned factors were subsequently analyzed using an Artificial Neural Network to minimize operational discrepancies and effectively optimize the variables. The ANN serves as an optimization technique based on mathematical models and algorithms, integrating learning and information processing systems that seek to replicate the cognitive processes of the human brain. The primary advantage of artificial neural networks is their inherent adaptability. The optimized variables generated results that were more consistent than those obtained with previous optimization efforts using a full factorial design. In confirmation with previous findings, it was ultimately determined that the significant variables were as follows: the mass of the adsorbent material (13 mg); the volume of the elution solvent (0.5 mL); the elution flow rate (1.75 mL min^−1^); and the composition of the eluent, expressed as the percentage of acetonitrile (ACN) in the H_2_O/ACN (*v*/*v*) mixture, specifically at 70% (*v*/*v*). Therefore, an artificial neural network with 27 data points, three of which were designated as central points, was employed to achieve recoveries ranging from 89% to 110% for all the compounds, with a maximum desirability approaching 90%, indicating an optimal value. Detailed information on the ANN model architecture, data partitioning, performance parameters, and desirability function is provided in the Supplementary Material under the section title Artificial Neural Network Optimization (Table S3 and Figure S2).

The performance of the optimized μSPE strategy was evaluated against previously reported extraction strategies for HAAs and related contaminants in food and environmental matrices (Table S2). QuEChERS-based approaches applied to soy products have been reported to provide recoveries between 62 and 93% [12], while MWCNT-based SPE achieved 80–110% in biomass samples [11], and MIL-53(Al)@cellulose paper devices yielded 86–114% for contaminants in water [22]. In comparison, the present method attained consistently high recoveries (84–100%) across ten HAAs in PBMAs, which represent a more complex matrix due to their protein, fat, and sugar contents. The poly(MAA-co-EDMA) monolithic sorbent minimized matrix effects and ensured quantitative elution, offering robustness and reproducibility superior to conventional SPE. Taken together, these findings position the proposed method as a reliable, efficient, and greener alternative for the quantification of HAAs in plant-based milk alternatives, while demonstrating performance that equals or surpasses the established methods in other food and environmental matrices.

### 3.2. Method Validation

The samples were spiked at several concentration levels in triplicate to assess analytical performance and determine recoveries via calibration curves, which were generated through linear regression analysis employing the least-squares method, with signal intensity (area) against the concentration of each HAA. Recovery experiments were performed using real PBMA samples, which were spiked with analytes at concentrations ranging from 0.005 to 100 µg L^−1^. The analytical figures of merit for the developed methodology are presented in Table 1.

The limits of detection and quantification for the 10 HAAs were estimated using Equations (1) and (2). The limits of detection and quantification values ranged from 0.01 to 0.04 μg L^−1^ and 0.02 to 0.05 μg L^−1^, respectively. The linear range was established for all analytes from concentrations close to the LOD up to 80 μg L^−1^. The coefficients of determination (r^2^) for all HAAs demonstrated a strong linear relationship, exceeding 0.996. Recovery rates varied between 84 % and 100 %. Precision assessments conducted both within day (intra-day) and across multiple days (inter-day) yielded relative standard deviation (RSD) values below 9% and 5%, respectively. Furthermore, enrichment factors (EFs) were found to range from 43 to 50.

Regarding matrix effects, it is important to note that, without prior sample treatment, direct injection into the mass spectrometer is not feasible due to the low analyte concentrations and high matrix complexity, which compromise reliable detection and may interfere with the analytical system. In this study, the implementation of a rigorous sample clean-up and extraction procedure substantially reduced the matrix effect from its initial 100% value to consistently very low values across all heterocyclic aromatic amines analyzed in the different plant-based beverages. Although minor differences remained, the residual matrix effects appear to correlate with the preparation variables considered (Table S4). Thus, the residual matrix-effect intervals obtained for each preparation are summarized as follows, highlighting the range observed across plant-based matrices: CSP/Soy (–5.42 to 5.44), CSP/Cashew (–5.50 to 5.25), CSP/Peanut (1.32 to 5.54), and CSP/Almond (0.20 to 2.00); H/Soy (–2.65 to 5.04), H/Cashew (–5.30 to 0.96), H/Peanut (–4.56 to 1.10), and H/Almond (1.50 to 4.70); HP/Cashew (–5.56 to 3.68), HP/Soy (–4.39 to 3.69), HP/Peanut (–5.40 to –0.85), and HP/Almond (–1.80 to 4.30); HS/Cashew (–5.07 to 4.67), HS/Almond (–4.20 to 5.10), HS/Peanut (–5.10 to 2.51), and HS/Soy (–2.32 to 3.56); and HSP/Almond (–5.10 to 5.30), HSP/Peanut (–5.56 to 3.87), HSP/Cashew (–5.35 to 4.07), and HSP/Soy (1.27 to 5.15). Further analysis of these findings suggests that, in the case of homemade beverages without sweetening or pasteurization (H), the residual matrix effect was more pronounced for certain amines, particularly MeIQx, PhIP, and MeAαC, which emerged as the most affected analytes. For the homemade sweetened (HS) beverages, the residual matrix effect was again detectable, with MeAαC showing the highest impact among the analytes, followed in frequency by PhIP and, to a lesser extent, MeIQx. In the homemade pasteurized (HP) beverages, the most frequent matrix effects were observed for PhIP, MeIQx, and IQ, with IQ showing a particularly marked response. These findings indicate that thermal treatment through pasteurization may enhance the residual effects for certain amines, especially IQ, likely due to compositional changes induced during heating that promote interactions with these analytes. For the homemade sweetened and pasteurized (HSP) beverages, matrix effects were observed for all heterocyclic aromatic amines, indicating a broader and more generalized impact compared to the other homemade preparations. The simultaneous application of sweetening and pasteurization appears to have a cumulative influence, likely enhancing interactions between beverage constituents and the HAAs, thereby increasing the overall susceptibility of all analytes to residual matrix effects. In the commercial sweetened and pasteurized (CSP) beverages, the residual matrix effect displayed a heterogeneous behavior depending on the plant source. Cashew-based beverages showed the most complex profile, with two groups of affected analytes that included DMIP, MeIQ, AαC, and MeαAC, evidencing a broader susceptibility to interference. Soy beverages, in contrast, were mainly influenced by PhIP and AαC, while peanut-based drinks were characterized by stronger effects on the carboline-type amines, particularly DMIP, IQ, and MeIQ. Almond beverages exhibited the lowest influence, with no marked matrix effects detected. Overall, within the homemade group, the combination of sweetening and pasteurization (HSP) resulted in a more generalized impact affecting all HAAs, with a distribution of effects across plant sources that closely resembled the pattern observed in the commercial sweetened and pasteurized (CSP) samples. Despite these differences, all residual matrix effects remained within acceptable RSD limits, confirming the robustness of the analytical method.

### 3.3. Evaluation of the Sustainability of the Analytical Methodology

**Figure 2:**
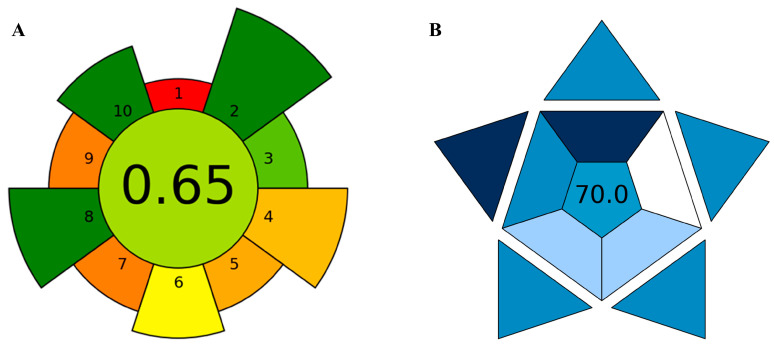
Assessment of the sustainability of the analytical procedure. (**A**) Evaluation of the proposed methodology using the AGREEprep metric. (**B**) Evaluation of the method’s practicality and applicability using the Blue Applicability Grade Index (BAGI).

The ecological sustainability of an analytical procedure represents a critical dimension of a technique’s overall suitability. To assess the environmental impact of the procedure, the AGREEprep metric was employed. The evaluation process using AGREEprep is characterized by its simplicity, and the user-friendly software interface facilitates efficient data input and interpretation of results. In the AGREEprep metric, a color-coded circular diagram is used to visualize the greenness of sample preparation procedures across ten principles of green analytical chemistry. Each segment represents one principle and is shaded from red (low compliance) to green (high compliance), with intermediate colors (e.g., yellow or orange) indicating moderate performance. The overall greenness score is shown in the center of the diagram, both numerically and through a corresponding color, providing a rapid visual assessment of the method’s environmental impact. An open-access version of this software can be obtained from the Internet [23]. For the proposed protocol, the following parameters were entered into the AGREEprep software: *1.* Sample preparation location: ex situ. *2.* Hazardous materials: no problematic reagents were used. *3.* Sustainability and renewability of reagents: over 75% of the solvents and materials used are renewable or reusable, including the poly(MAA-co-EDMA) sorbent. *4.* Waste generation: approximately 5 mL per sample. *5.* Sample volume: 10 mL. *6.* Sample throughput: approximately eight samples per hour. *7.* Integration and automation: four steps, with semi-automated processes. *8.* Energy consumption: around 2.5 W per extraction (centrifuge and vacuum pump), classified as low. *9.* Post-preparation setup for analysis: directly coupled to LC-MS/MS. *10.* Operator safety: no hazardous reagents or direct exposure involved. The resulting weighted composite score of 0.65 is relatively high compared to conventional SPE or LLE procedures (with typical AGREEprep scores ranging from 0.30 to 0.50). This enhanced score is primarily attributed to the absence of toxic solvents, low waste generation, and the reuse of sorbent material. The application of this metric is depicted in Figure 2A, which indicates a result of 0.65, signifying a high level of sustainability.

Additionally, the use of an index designed to efficiently evaluate the practicality and applicability of a developed analytical method is recommended. For this purpose, the BAGI metric was utilized to quantify the functionality of the analytical method [24]. Taking into account the number of analytes (10 HAAs), the sample throughput (5–12 samples per hour), the need for preconcentration (µSPE), the level of automation (semi-automated collector), and the type of instrumentation used (UHPLC–MS/MS), the results presented in Figure 2B yielded a score of 0.70, indicating that this methodology is both adequate and functional.

### 3.4. Presence and Quantification of HAAs in PBMAs

**Figure 3:**
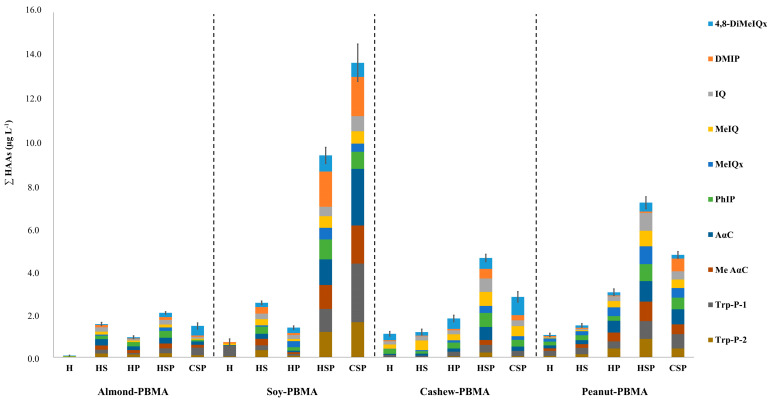
Analysis of HAAs in plant-based milk alternatives (PBMAs). Sample types: (**H**) Homemade, unsweetened and unpasteurized; (**HS**) Homemade, sweetened and unpasteurized; (**HP**) Homemade, unsweetened and pasteurized; (**HSP**) Homemade, sweetened and pasteurized; and (**CSP**) Commercial, sweetened and pasteurized.

The analysis of heterocyclic aromatic amines across all samples revealed a clear influence of beverage type, sugar addition, and pasteurization on HAA occurrence and concentration (Figure 3, Table S5).

Overall, total HAA concentrations ranged from non-detectable levels to 13.66 μg L^−1^, with commercial samples presenting higher levels (1.43–13.66 μg L^−1^) compared to homemade beverages (0.09–9.36 μg L^−1^). This difference was statistically significant at the 95% confidence level (*p* ≈ 0.02; 95% Confidence Interval: 0.7–6.5 μg L^−1^), confirming that commercial samples consistently exhibited higher HAA concentrations. Similar *p*-values and 95% confidence intervals have been calculated for all key comparisons to ensure a robust and accurate interpretation of the data.

Almond-based homemade beverages that were neither sweetened nor pasteurized did not contain DMIP, IQ, MeIQx, AαC, Me AαC, Trp-P-1, or Trp-P-2, and pasteurization alone did not result in the formation of MeIQx. In contrast, soy- and peanut-based beverages were consistently more affected, with most samples containing multiple HAAs. Cashew-based drinks showed intermediate behavior, with Me AαC detected only when both sugar-addition and pasteurization processes were applied. Previous studies have indicated that heterocyclic aromatic amines can be generated when free amino acids and reducing sugars coexist [12,37].

When the most abundant HAA in each treatment group was examined, 4,8-DiMeIQx was predominant in unsweetened/unpasteurized almond (0.03 μg L^−1^) and cashew (0.30 μg L^−1^) beverages, whereas Trp-P-1 was most abundant in soy (0.49 μg L^−1^) and peanut (0.24 μg L^−1^) beverages. The highest individual HAA concentrations were observed in soy beverages after sweetening and pasteurization (DMIP, 1.63 μg L^−1^) and in peanut beverages under the same conditions (AαC, 0.96 μg L^−1^). In commercial products, Trp-P-1 in soy beverages (2.72 μg L^−1^) and AαC in peanut beverages (0.70 μg L^−1^) represented the most abundant HAAs, highlighting soy and peanut drinks as the main contributors to dietary HAA exposure.

Considering the lower protein and macronutrient content of almond- and cashew-based beverages, the HAAs formed in these matrices were mainly of the thermal group. Conversely, soy- and peanut-based drinks presented the higher risks associated with pyrolytic HAAs, suggesting a greater potential contribution to long-term dietary exposure. These quantitative findings provide essential data for subsequent risk assessment and the calculation of incremental lifetime cancer risk values.

### 3.5. Influence of Nutritional Composition, Sugar Addition, and Thermal Processing on the Concentration of HAAs

**Figure 4:**
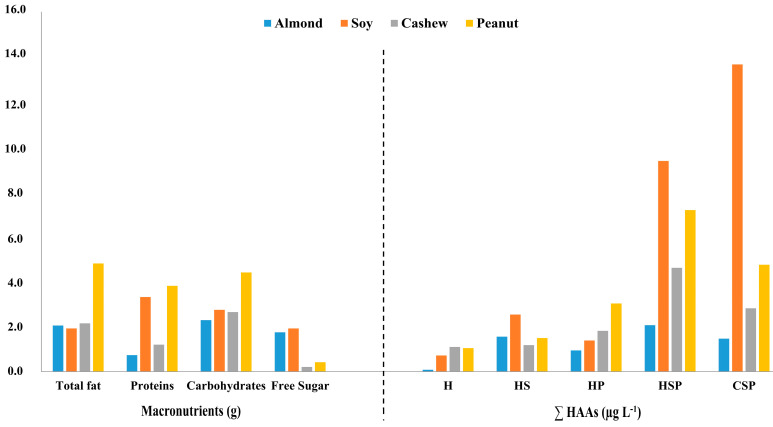
Correlation between HAA concentrations and the compositional characteristics of each plant-based beverage.

The proposed methodology has been demonstrated to be adequate, straightforward, robust, and suitable for routine analytical applications. Plant-based milk alternatives exhibit a notable nutritional profile, being particularly abundant in B vitamins, dietary fiber, protein, and healthy fats, while simultaneously being free from lactose and cholesterol [38,39]. These advantages encourage consumers to substitute animal products with these plant-based alternatives, allowing them to obtain essential nutrients necessary for growth and development. Research indicates that the conditions and nutritional attributes of these plant-based beverages—particularly the presence of reducing sugars and free amino acids—create an environment that is favorable for the formation of HAAs when exposed to sweetening processes, heat treatments, or a combination of both. This establishes a direct correlation not only with the processing methods employed for these beverages, but also with the chemical precursors responsible for the generation of these contaminants, which arise from their nutritional parameters [40]. This finding highlights the direct relationship between the concentration of identified HAAs and the nutritional composition of each plant-based beverage, as illustrated in Figure 4.

**Figure 5:**
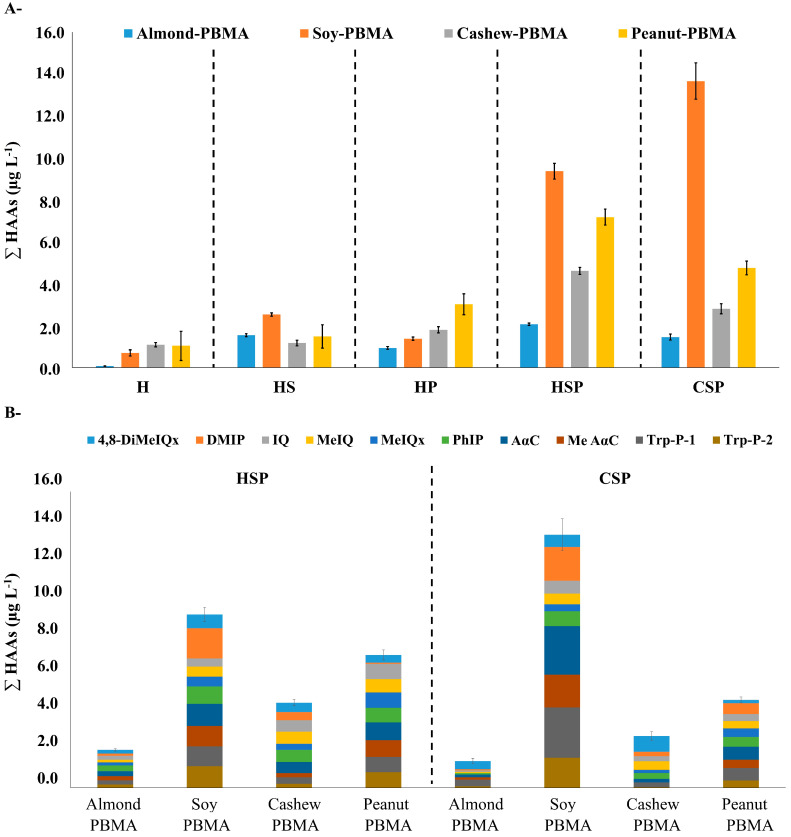
(**A**) Synergistic effects of sugar addition and thermal treatment on the formation of HAAs in processed beverage matrices. (**B**) Concentration patterns of HAAs observed in both commercially produced and homemade beverages subjected to sweetening and pasteurization.

In this context, higher total concentrations of HAAs were quantified in matrices with elevated protein content. Significant differences in total HAAs were also identified between commercial and homemade samples, particularly in those that underwent pasteurization. The increase in HAAs was particularly significant in beverages with added sugars that were simultaneously pasteurized, compared to those that underwent only heat treatment, contained solely added sugars, or did not undergo any manufacturing process. This finding suggests a synergistic interaction among the total concentration of HAAs, the intrinsic properties of the matrix, and the combined effects of sweetening and heat treatment. Overall, both commercial beverages and those that were sweetened and pasteurized exhibited similarly concerning total amounts of HAAs, as shown in Figure 5.

### 3.6. Application of Principal Component and Cluster Analyses

This section of the study focused on an exploratory analysis, utilizing multivariate statistical methods as the main analytical approach. Initially, the data obtained from previous tests were analyzed using an unsupervised method, namely Principal Component Analysis (PCA). Subsequently, cluster analysis was employed to find patterns in the identity and concentrations of HAAs determined in the plant-based beverage samples, allowing for their classification into groups based on the similarity or origin of these concentrations. The aim was to elucidate the presence in PBMAs of the HAAs generated during their manufacturing process, thereby facilitating the development of a model that ensures the authenticity of these correlations and the final products.

The principal components analysis revealed that two principal components accounted for up to 87.3% of the total variance in the assessment of total concentrations of heterocyclic aromatic amines across all plant-based milk alternatives. Specifically, Component 1 (CP1) explained 75.5% of the variance, while Component 2 (CP2) accounted for 11.8%. CP1 was primarily influenced by most of the heterocyclic aromatic amines examined, which were grouped within matrices characterized by the combination of added sugar and pasteurization. This suggests a direct correlation with CP1. Conversely, matrices exhibiting lower concentrations of HAAs were associated with CP2, particularly those vegetable beverages that underwent no processing (neither sweetening nor pasteurization) or were subjected to only one of these processes. The PCA further substantiated the statistical relationships between the concentrations of HAAs detected and the nutritional or manufacturing variables pertinent to each vegetable beverage. In the case of homemade samples, although a comprehensive analysis of variability in plant-based beverages typically requires a larger number of principal components, this study utilized a simplified model that employed only two components (PC1 and PC2) for each type: almond, soy, cashew, and peanut. Despite this reduction, a significant proportion of the total variance was still considered: 99.9% for almond beverages (84.5% from PC1 and 15.4% from PC2); 97.1% for soy beverages (91.4% from PC1 and 5.6% from PC2); 89.2% for cashew beverages (entirely from PC1); and 93.7% for peanut beverages (entirely from PC1), with minor contributions from PC2 (9.2% and 6.1%, respectively). In summary, unsweetened and/or unpasteurized vegetable beverages did not exhibit a significant dominance of any group of HAAs, and their distribution in each principal component analysis score scatter plot was similar. In contrast, polar HAAs, including DMIP, IQ, MeIQ, MeIQx, 4,8-DiMeIQx, and PhIP, were found to be more prevalent in food matrices that underwent pasteurization, as demonstrated by the almond-, cashew-, and soy-based drinks. Conversely, the pasteurization of peanut-based beverages resulted in a heterogeneous mixture of HAAs, lacking clear differentiation. Further information is provided in Figure S3.

Thermal processes conducted under conditions of elevated protein levels or significant concentrations of free sugars promoted the formation of polar HAAs. When these processes are combined, there is a marked exponential increase in the total concentration of the HAAs analyzed. A comparable phenomenon is observed in plant-based beverages that exhibit a high protein content relative to their total sugar content, as observed in soy- or peanut-based drinks. In the latter case, the higher concentration of total fats resulted in an increased concentration of analytes when sweetening and pasteurization are applied concurrently. In instances where free sugars are predominant, such as in almond- or cashew-based beverages, trends suggested a general increase in polar HAAs. This increase may be attributed to the sweetening of these beverages or their exposure to thermal processing.

Additionally, the classification of samples according to the type of grain or bean revealed significant differences among the analyzed beverages. This analysis not only elucidated the identified groups, but also recognized trends that indicated a synergistic effect on the total concentration of contaminants when thermal treatment is applied to sugar-sweetened beverages. This phenomenon was consistently observed in both the commercially produced and homemade samples In these preliminary studies, it was evident that the macronutrient composition of each beverage, along with the production methods employed, significantly influenced the differentiation and classification of these drinks into distinct categories. The results of this study demonstrate that the application of thermal processing, in conjunction with the addition of a sweetener such as sugar, leads to an increase in the concentration of heterocyclic amines, particularly in plant-based beverages with an elevated protein content. In these specific beverages, both categories of HAAs demonstrate exponential growth. On the other hand, within the central region of the principal component analysis score plot, there exists a cluster predominantly composed of matrices where a synergistic interaction between sugar and heat is absent. These matrices either contain only free sugars as their premodifying macronutrients or have a lower protein content, which is necessary for the enrichment of these compounds (in contrast to soy- and peanut-based beverages). However, in cases where sweetening was combined with pasteurization, an increase in the final concentration of heterocyclic aromatic amines was observed. Further information is provided in Figure S4.

## 4. Evaluation of Health Risks Associated with the Consumption of Plant-Based Milk Alternatives

### 4.1. Estimation of Daily Intake and Chronic Daily Intake

In the context of the ten analyzed HAAs, the assessment of exposure and potential carcinogenic effects necessitates an understanding of the quantities of these contaminants consumed via dietary sources. These data are crucial for the computation of estimated daily intake (EDI, as represented in Equation (4)) and chronic daily intake (CDI, as defined in Equation (5)), both of which quantify the amount of a substance that an individual assimilates within a single day.

At present, there is a lack of comprehensive documentation regarding the average consumption of plant-based beverages. The recommended daily intake of dairy products is established at two to three servings, with each serving ranging from 200 to 250 mL [23]. The amount of heterocyclic aromatic amines ingested from the consumption of vegetable drinks was determined, considering the recommended serving sizes of dairy products. Based on the previously established correlations in this study, vegetable beverages that were characterized by elevated levels of protein and free sugars, particularly when subjected to thermal processing and the addition of sweeteners, tended to demonstrate an increased concentration of HAAs. This phenomenon results in consumption levels that significantly exceed the daily intake limit of 1 μg for HAAs, as stipulated by the European Council [14]. The findings of this study, alongside the high chronic daily intake levels, indicate a substantial elevation in dietary exposure to HAAs. This raises significant concerns regarding the associated risks linked to the concentration of HAAs in both commercially produced and homemade beverages, representing a significant case of documented risk. The estimated daily intake of HAAs (mg day^−1^) is derived from the recommended serving sizes of PBMAs. This evaluation considers the chronic daily intake (CDI) levels established for these beverages, assuming a lifetime consumption of plant-based beverages equivalent to one recommended daily serving of dairy products (mg kg^−1^ day^−1^). The chronic daily intake of plant-based beverages has been established for various age groups, including 5, 13, and 84 years of age. From the findings, CDI varied significantly depending on the type of plant-based milk (almond, soy, cashew, peanut) and the specific processing method (H, HS, HP, HSP, CSP). Generally, these values decreased as exposure time increased, suggesting a reduced consumption of these products with advancing age. Soy-based beverages consistently exhibited higher CDI values compared to other types of beverages, particularly for the 5-year-old age group and in the CPS scenario. In contrast, almond-based beverages demonstrate the lowest CDI across all scenarios and exposure times, while cashew- and peanut-based beverages fall within the intermediate range, although variations occur depending on the type of processing.

### 4.2. Incremental Lifetime Cancer Risk: Carcinogenicity Risk Assessments by Type of Plant-Based Beverage

The incremental lifetime cancer risk (ILCR) quantifies the likelihood that an individual will develop cancer over their lifetime because of exposure to a carcinogen. The mean total concentrations of heterocyclic aromatic amines in the samples represented the worst-case scenario concerning the safety of PBMAs as consumable food products. In each case, dietary exposure to HAAs was calculated using a validated cancer slope factor (CSF), with the assumption of an additive carcinogenic effect resulting from co-exposure to HAAs. These data were used to calculate Equation (6), allowing for the determination of the carcinogenic risk associated with each beverage based on its recommended serving size, which ranged from one to five portions (with one portion defined as 200 mL of PBMAs), along with the chronic daily intake levels established for these beverages. The results revealed that for most plant-based beverages, the ILCR values were classified within or very near the intolerable range, even with the consumption of a single serving in certain instances, indicating a potential health risk associated with the consumption of these products. The results are illustrated in Table 2. The health risk assessment revealed that, although the incremental lifetime cancer risk values remained below the regulatory threshold of concern (10^−4^), several scenarios approached or even bordered on 10^−5^, particularly for frequent consumers of plant-based milk alternatives. This pattern indicates a moderate but non-negligible carcinogenic risk, reflecting the detectable presence of heterocyclic aromatic amines in these beverages. These findings highlight the importance of continued monitoring and more comprehensive toxicological evaluations of plant-based milk alternatives to ensure safe consumption, particularly given their increasing role as mainstream dietary choices in modern nutrition.

## 5. Conclusions

This research investigated the quantification of ten heterocyclic aromatic amines in both homemade and commercially produced vegetable beverages derived from almonds, soy, cashews, and peanuts. The analysis was conducted utilizing an innovative μSPE protocol in conjunction with a UHPLC-MS/MS determination, which exhibited a robust performance. The methodology yielded a linear range spanning from 0.01 to 80 μg L^−1^, with correlation coefficients approximating 0.996, recovery rates ranging from 84% to 100%, and enrichment factors reaching up to 50 times. The sustainability metrics associated with HAAs in vegetable drinks were also evaluated. Through the application of Principal Component Analysis and Cluster Analysis, a direct correlation was identified between the concentrations of the detected HAAs, the macronutrient content, and the sweetening and thermal processing methods, or their combinations, employed for each sample. The carcinogenic risk associated with each beverage was assessed based on its recommended serving size, indicating that the ILCR values for most plant-based milk alternatives fell within or near the intolerable range, suggesting a potential health concern even from a single serving. However, it is important to contextualize these findings within realistic dietary exposure scenarios. The ILCR estimates assume daily consumption of these beverages at the highest observed HAA concentrations, whereas typical dietary patterns involve a variety of foods, which could dilute the potential risk from any single source. Therefore, although the presence of carcinogenic HAAs is notable and warrants attention, the actual risk for most consumers is likely lower than the extreme-case scenarios might suggest. This study represents the first report of HAAs in vegetable drink samples worldwide, thereby providing significant insights into their prevalence. Furthermore, since comprehensive ILCR data for HAAs in food matrices remain largely unavailable—primarily due to a lack of validated oral slope factors—this work addresses this gap by contributing novel risk estimation data for ten HAAs. This is particularly relevant given that such evaluations are scarce in both animal- and plant-based dairy alternatives. Consequently, the findings of this study highlight the critical need for vigilant monitoring of HAA concentrations in widely consumed foods such as plant-based milk, particularly as alternatives to traditional animal-derived milk products.

Future research could incorporate a formal sensitivity analysis to assess the ILCR results based on varying assumptions about serving size and consumption frequency, thereby improving the evaluation of potential risks. Moreover, broader surveys across different brands, formulations, and countries are also needed to capture variability in contaminant occurrence and exposure scenarios. In parallel, efforts should focus on developing mitigation strategies to reduce HAA formation, such as optimizing processing conditions, applying alternative pasteurization technologies, or exploring the use of natural inhibitors. Together, these approaches will strengthen the connection between analytical innovation and preventive public health strategies, ultimately supporting evidence-based regulation and safer consumption of plant-based beverages.

## Figures and Tables

**Table 1:** Analytical figures of merit for the developed methodology.

Compounds	4,8-DiMeIQx	DMIP	IQ	MeIQ	MeIQx	PhIP	AαC	MeAαC	Trp-P-1	Trp-P-2
**LOD (µg L^−1^)**	0.02	0.02	0.04	0.02	0.01	0.02	0.01	0.01	0.01	0.02
**LOQ (µg L^−1^)**	0.04	0.03	0.05	0.04	0.03	0.04	0.03	0.02	0.03	0.03
**Lineal Range (µg L^−1^)**	0.04–80	0.03–80	0.05–80	0.05–80	0.03–80	0.04–80	0.03–80	0.02–80	0.03–80	0.03–80
**r^2^**	0.996	0.999	0.996	0.999	0.996	0.998	0.996	0.997	0.996	0.998
*R* (%), *n* = 3 *	97 **± 5**	84 **± 3**	89 **± 4**	98 **± 3**	91 **± 4**	95 **± 2**	95 **± 3**	85 **± 4**	100 **± 3**	88 **± 1**
**Intra-day Precision** **(RSD (%), *n* = 3**	4.65 **± 0.42**	1.94 **± 0.17**	3.89 **± 0.35**	2.13 **± 0.20**	3.67 **± 0.33**	1.02 **± 0.10**	3.24 **± 0.30**	4.12 **± 0.37**	3.89 **± 0.35**	2.11 **± 0.20**
**Inter-day Precision** **(RSD (%), *n* = 3**	9.01 **± 1.35**	2.92 **± 0.44**	4.90 **± 74**	3.83 **± 0.57**	4.42 **± 0.66**	1.76 **± 0.26**	4.52 **± 0.68**	6.51 **± 0.9**	4.35 **± 0.65**	3.36 **± 0.50**
***EF***	49	49	43	50	46	48	46	46	49	48

* ±Standard deviation.

**Table 2:** Assessment of incremental lifetime cancer risk for plant-based beverages.

Almond-PBMA	1 Service			2 Services			3 Services			4 Services			5 Services		
	5	13	84	5	13	84	5	13	84	5	13	84	5	13	84
H															
HS															
HP															
HSP															
CSP															
**Soy-PBMA**	**1 Service**			**2 Services**			**3 Services**			**4 Services**			**5 Services**		
	5	13	84	5	13	84	5	13	84	5	13	84	5	13	84
H															
HS															
HP															
HSP															
CSP															
**Cashew-PBMA**	**1 Service**			**2 Services**			**3 Services**			**4 Services**			**5 Services**		
	5	13	84	5	13	84	5	13	84	5	13	84	5	13	84
H															
HS															
HP															
HSP															
CSP															
**Peanut-PBMA**	**1 Service**			**2 Services**			**3 Services**			**4 Services**			**5 Services**		
	5	13	84	5	13	84	5	13	84	5	13	84	5	13	84
H															
HS															
HP															
HSP															
CSP															
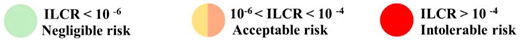															

H: Homemade sample, unsweetened and unpasteurized; HS: Homemade sample, sweetened and unpasteurized; HP: Homemade sample, unsweetened and pasteurized; HSP: Homemade sample, sweetened and pasteurized; CSP: Commercial sample, sweetened and pasteurized. Age groups: 5, 13, and 84 years of age. Serving sizes: 1 service (200 mL), 2 services (400 mL), 3 services (600 mL), 4 services (800 mL), and 5 services (1000 mL).

## Data Availability

The original contributions presented in this study are included in the article/Supplementary Material. Further inquiries can be directed to the corresponding author(s).

## Supplementary Materials

The following supporting information can be downloaded at https://www.mdpi.com/article/10.3390/foods14193295/s1: Figure S1: Schematic representation of the solid-phase microextraction procedure followed by high-performance liquid chromatography coupled with tandem mass spectrometry (μSPE-UHPLC-MS/MS); Figure S2: Desirability surfaces obtained by ANN modelling as a function of: A. elution solvent volume and mass of adsorbent material; B. elution flow rate and mass of adsorbent; C. elution solvent composition and mass of adsorbent; D. elution flow rate and elution solvent volume; E. elution solvent composition and elution solvent volume; and F. elution solvent composition and elution flow rate. In each figure, the third factor was maintained at an optimal value; Figure S3: Principal component analysis (PCA) conducted for different PBMAs. Panels A, C, E, and G display the PCA scores scatter plots for almond, soy, cashew, and peanut-based milk, respectively. Panels B, D, F, and H illustrate the loading plots of PCA for each respective type of milk alternative; Figure S4: Scores plot obtained from principal component analysis for all PBMAs. B. Loading plot resulting from the PCA conducted on PBMAs. C. Hierarchical clustering dendrogram constructed based on the concentrations of heterocyclic aromatic amines and the classification of PBMAs; Table S1: List of heterocyclic aromatic amines with their nomenclature, chemical formula, structure and IUPAC name; Table S2: Comparative Analysis and Sustainability Assessment of Associated Extraction Techniques; Table S3: ANN experimental design; Table S4: Residual matrix effects for each HAA across plant-based beverage preparations; Table S5: Total and individual concentrations of HAAs in the plant-based milk alternatives analyzed.

## Abbreviations

AGREEprep, Analytical Greenness metric for sample preparation; ANNs, Artificial Neural Networks; BAGI, Blue Applicability Grade Index; CDI, Chronic Daily Intake; CSF, Cancer Slope Factor; CSP, Commercial sample, sweetened and pasteurized; EDI, Estimated Daily Intake; EF, Enrichment Factor; H, Homemade sample, unsweetened and unpasteurized; HAAs, Heterocyclic aromatic amines; HP, Homemade sample, unsweetened and pasteurized; HS, Homemade sample, sweetened and unpasteurized; HSP, Homemade sample, sweetened and pasteurized; HTST, High-temperature short-time; ILCR, Incremental Lifetime Cancer Risk analysis; LOD, Limit of detection; LOQ, Limit of quantification; LTLT, Low-temperature long-time; PBMAs, Plant-based milk alternatives; PCA, Principal component analysis; R, Recovery percentage; UHPLC-MS/MS, Ultra-high-performance liquid chromatography coupled with tandem mass spectrometry; UHT, Ultra-high temperature; μSPE, Solid-phase microextraction.
