# Supplementary material for: Quantitative Analysis and Health Risk Assessment of Heterocyclic Aromatic Amines in Plant-Based Milk Beverages
Source: Foods. 2025 Sep 23;14(19):3295. doi: 10.3390/foods14193295 (PMC12523737; doi:10.3390/foods14193295)
Supplement: Supplementary file 1 [file foods-14-03295-s001.zip › foods-3808482-supplementary.pdf]

Supporting Information

Table S1. List of heterocyclic aromatic amines with their nomenclature, chemical formula, structure and IUPAC name.

Nomenclature	*Chemical formula*	*Structure*	*IUPAC name*
4,8-DiMeIQx	$C_{12}H_{13}N_5$	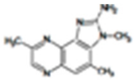	2- amino-3,4,8-trimethylimidazo[4,5-f]quinoxaline
DMIP	$C_8H_{10}N_4$	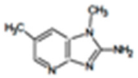	2-amino-1,6-dimethylimidazo[4,5-b]pyridine
IQ	$C_{11}H_{10}N_4$	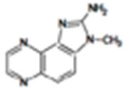	2-amino-3-methylimidazo[4,5-f]quinoline
MeIQ	$C_{12}H_{12}N_4$	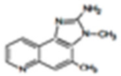	2-amino-3,4-dimethylimidazo[4,5-f]quinoline
MeIQx	$C_{11}H_{11}N_5$	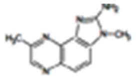	2-amino-3,8-trimethylimidazo[4,5-f]quinoxaline
PhIP	$C_{13}H_{12}N_4$	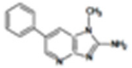	2-amino-1-methyl-6-phenylimidazo[4,5-b]pyridine
AαC	$C_{11}H_9N_3$	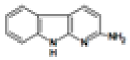	2-amino-9H-pyrido[2,3-b]indole
Me AαC	$C_{12}H_{11}N_3$	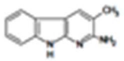	2-amino-3-methyl-9H-pyrido[2,3-b]indole
Trp-P-1	$C_{13}H_{13}N_3$	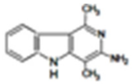	3-amino-1,4-dimethyl-5H-pyrido[4,3-b]indole

Trp-P-2	$C_{12}H_{11}N_3$	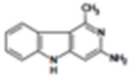	3-amino-1-methyl-5H-pyrido[4,3-b]indole

Table S2. Comparative Analysis and Sustainability Assessment of Associated Extraction Techniques

**Sample**	**Methodology**		**Compounds** (number of compounds)	**Performance**		**Green Metrics**		**Ref.**
	Sample procedure	Analysis technique		(R (%))	(EF)	AGREEprep	BAGI	
Biomass from cigarette and tobacco combustion	MWCNT-based SPE	UHPLC-MS/MS	HAAs (10)	80–110	No informed	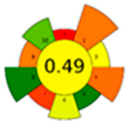 0.49 Points	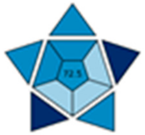 72.5 Points	[11]
Soy products	QuEChERS	UHPLC-MS/MS	HAAs (12)	62–93	No informed	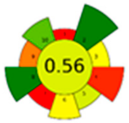 0.56 Points	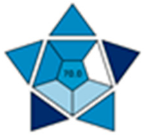 70 Points	[12]
Human plasma and urine	SPME	MS (QTRAP, nano-ESI)	Ketoprofen<br>Flurbiprofen	Plasma: 82–107<br>Urine: 89–109	221-348	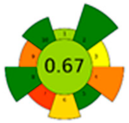 0.67 Points	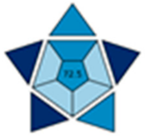 72.5 Points	[13]

Environmental water	MIL-53(Al)@ cellulose paper device	HPLC-DAD	Neonicotinoids (5)	86–114	10	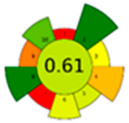<br>0.61 Points	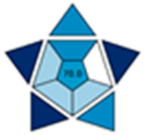<br>70 Points	[22]
Water	poly(MAA-co-EDMA) monolithic material-based SPE	UHPLC-MS/MS	HAAs (3)	98–100	95–99	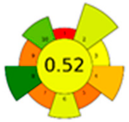<br>0.52 Points	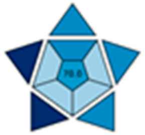<br>70 Points	[20]
Plant-based milk alternatives (PBMAs)	poly(MAA-co-EDMA) monolithic material-based SPE	UHPLC-MS/MS	HAAs (10)	84-100	47-50	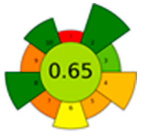<br>0.65 Points	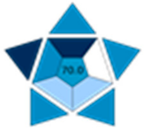<br>70 Points	**This Work**

3.1. Optimization of Retention and Elution Conditions

Artificial Neural Network Optimization

Artificial neural networks (ANNs) represent a robust approach for modeling nonlinear systems without necessitating prior knowledge of the underlying analytical functions. In the present research, a radial basis function (RBF) ANN was developed utilizing the surface response optimization by artificial neural networks (SRO_ANN) implemented in the MATLAB toolbox [1]. Model performance was assessed using twenty-seven experimental datasets. The input variables comprised mass of sorbet material (mg), elution solvent volume (mL), elution flow rate (mL min^{-1}), and eluent composition (expressed as the percentage of acetonitrile in water, v/v, % ACN), while the response variable was defined as the recovery percentage of HAAs. The experimental design is detailed in Table S2.

The dataset was randomly partitioned into training (70%), testing (15%), and validation (15%) subsets. Various network architectures featuring between one and ten hidden neurons and a single output neuron were evaluated. Training of the ANN was conducted in MATLAB R2022b employing the Levenberg–Marquardt backpropagation algorithm to effectively model the nonlinear relationships between inputs and output. Subsequent optimization of the ANN-modeled data was carried out using a desirability function with the objective of maximizing analyte recovery. The optimal conditions (desirability index D = 0.88) were identified as a mass of sorbent of 13 mg, elution solvent volume of 0.5 mL, elution flow rate of 1.75 mL·min^{-1}, and an eluent composition of 70% ACN in water. Under these optimized parameters, recovery rates ranged from 89% to 110%. Experimental validation of the ANN-predicted optimal conditions demonstrated no statistically significant differences between predicted and observed recoveries, as confirmed by a Student's t-test ($t < 2.92$, $p = 0.05$, $n = 3$). Desirability response surfaces for pairs of factors, with the remaining variable held constant at its optimal value, are presented in Figure S2.

Table S3. ANN experimental design.

	Factors				*HAAs - Response*[a]									
**Datasets**	**Mass of sorbent material (mg)**	**Elution solvent volume (mL)**	**Elution flow rate (mL min^{-1})**	**Elution solvent composition (% ACN)**	**4,8-DiMeIQx**	**DMIP**	**IQ**	**MeIQ**	**MeIQx**	**PhIP**	**AαC**	**Me AαC**	**Trp-P-1**	**Trp-P-2**
1	15	0.3	2.25	75	95.7	77.4	98.1	63.1	98.1	69.6	95.0	86.0	93.7	84.7
2	15	0.1	4	100	41.9	32.5	43.8	51.5	43.8	30.0	32.1	32.5	39.9	41.3
3	5	0.1	4	100	32.1	35.4	31.6	46.3	27.7	36.2	27.3	19.9	31.2	24.2

4	15	0.5	0.5	50	73.6	47.9	91.4	91.5	75.9	79.9	87.8	54.9	93.1	52.1
5	5	0.1	0.5	50	73.1	70.6	73.4	55.3	75.1	68.2	56.3	74.3	51.4	74.4
6	10	0.1	2.25	75	95.9	105.5	106.9	96.6	83.3	95.1	98.9	101.4	96.0	99.8
7	10	0.3	0.5	75	94.7	87.4	80.4	95.5	85.8	100.1	80.0	100.2	95.5	105.2
8	15	0.5	4	50	58.3	72.8	70.9	92.4	95.1	60.6	81.7	51.6	91.8	32.2
9	5	0.1	0.5	100	55.9	39.7	56.5	47.5	65.1	40.0	42.2	48.2	54.0	59.3
10	5	0.5	0.5	50	34.7	55.7	78.8	62.8	65.4	77.5	70.1	73.0	51.6	75.9
11	10	0.3	2.25	75	102.8	96.2	91.3	97.7	106.5	100.1	97.3	95.0	99.7	100.7
12	5	0.5	0.5	100	31.1	20.0	51.1	46.7	35.4	57.9	61.8	58.3	64.6	62.3
13	5	0.5	4	50	94.4	39.2	67.7	49.6	64.0	56.7	86.8	47.9	46.8	53.7
14	5	0.5	4	100	54.1	35.8	49.1	43.7	43.6	42.7	44.6	31.7	37.5	27.3
15	15	0.5	4	100	44.1	39.9	53.4	64.1	41.8	61.1	43.3	43.6	64.8	46.8
16	5	0.1	4	50	57.6	20.8	53.4	54.8	38.0	28.8	37.7	48.7	32.8	45.2
17	10	0.5	2.25	75	97.7	98.3	97.1	96.4	91.7	95.6	99.1	102.6	104.0	101.4
18	10	0.3	2.25	100	68.5	79.0	55.7	93.5	99.1	81.8	92.8	100.6	101.3	97.7
19	15	0.5	0.5	100	73.8	55.7	80.6	77.0	81.0	93.5	94.1	99.7	101.4	100.1
20	10	0.3	2.25	75	81.4	88.4	82.1	83.1	82.5	96.5	97.1	97.4	99.6	98.5

21	15	0.1	0.5	50	103.5	96.0	94.8	94.8	92.9	73.8	41.8	45.7	59.1	49.1
22	15	0.1	0.5	100	78.8	83.3	72.9	76.8	69.9	78.8	92.2	108.2	91.9	104.0
23	10	0.3	2.25	50	84.2	97.1	97.4	90.7	89.2	75.8	76.7	83.4	90.0	78.2
24	5	0.3	2.25	75	82.4	61.8	61.7	67.2	70.2	68.4	81.5	85.1	65.5	85.2
25	10	0.3	2.25	75	96.8	98.9	97.7	97.3	97.5	100.6	94.6	98.8	98.5	100.9
26	15	0.1	4	50	59.9	23	54.5	73.5	56	47.4	26.1	27.6	62.9	18.4
27	10	0.3	4	75	22.2	10.7	0.3	0.4	12.6	0.3	10.3	20.1	20.6	12.3

[a] Response represents the recovery percentage of each analyte.

Table S4. Residual matrix effects for each HAA across plant-based beverage preparations

	[a]H				[b]HS				[c]HP				[d]HSP				[e]CSP			
	**Almond**	**Soy**	**Cashew**	**Peanut**	**Almond**	**Soy**	**Cashew**	**Peanut**	**Almond**	**Soy**	**Cashew**	**Peanut**	**Almond**	**Soy**	**Cashew**	**Peanut**	**Almond**	**Soy**	**Cashew**	**Peanut**
**4,8-DiMeIQx**	2.80	4.62	-1.50	-2.15	2.00	0.27	-2.63	-3.44	2.60	-0.91	-1.50	-1.35	5.30	5.15	2.45	-5.56	2.00	3.30	1.28	4.99
**DMIP**	2.80	3.92	-2.42	-1.10	-3.00	3.56	-1.22	-4.21	-1.70	1.21	-4.52	-2.43	-3.10	1.27	4.07	1.31	1.40	-4.73	-5.50	1.32
**IQ**	3.60	0.20	0.96	-4.56	3.20	-1.23	-3.80	-3.32	4.30	3.27	-4.18	-3.77	-5.10	1.27	-5.35	3.87	0.50	0.14	-0.42	5.42
**MeIQ**	3.50	2.48	-2.04	-1.93	3.20	1.05	-4.48	-0.41	2.70	-3.80	-3.50	-4.50	-4.20	2.70	-3.65	-3.99	0.20	2.36	-4.76	5.54
**MeIQx**	2.40	5.04	-4.34	-4.33	1.40	2.06	4.67	-3.11	-1.80	3.39	-3.47	-0.85	5.00	2.92	3.17	-2.05	1.00	-0.40	2.53	3.88
**PhIP**	4.70	-2.65	-4.64	-2.52	-4.20	-2.32	2.96	2.51	-1.80	3.69	-3.22	-5.40	-3.50	4.68	2.68	-2.02	1.80	-5.42	1.22	3.61
**AαC**	1.50	-2.50	-1.17	-3.18	4.70	1.31	-0.62	-2.58	1.90	-4.39	-1.47	-1.62	5.00	3.63	1.42	2.70	1.00	5.44	4.76	3.82
**Me AαC**	2.40	-1.07	0.42	-3.40	5.10	0.52	-5.07	-2.74	2.30	1.36	3.68	-2.36	-3.90	5.10	-3.62	-3.22	1.60	3.77	5.25	4.55
**Trp-P-1**	2.90	3.63	-5.30	-2.38	-3.30	2.17	-1.55	-5.10	-1.70	1.81	-5.56	-4.47	5.10	3.32	2.22	-1.46	0.30	-3.86	4.16	4.39
**Trp-P-2**	1.70	0.12	-2.59	-4.41	2.00	-1.48	-1.68	2.06	2.40	2.42	-3.61	-3.79	3.30	3.02	-0.49	1.82	1.90	5.22	4.20	2.68

[a](**H**) Homemade sample, unsweetened and unpasteurized; [b](**HS**) Homemade sample, sweetened and unpasteurized; [c](**HP**) Homemade sample, unsweetened and pasteurized; [d](**HSP**) Homemade sample, sweetened and pasteurized; [e](**CSP**) Commercial sample, sweetened and pasteurized.

Table S5. Total and individual concentrations of HAAs in the plant-based milk alternatives analyzed.

HAAs (µg L^{-1})	Almond-PBMA					Soy-PBMA					Cashew-PBMA					Peanut-PBMA				
	**H**[a]	**HS**[b]	**HP**[c]	**HSP**[d]	**CSP**[e]	**H**	**HS**	**HP**	**HSP**	**CSP**	**H**	**HS**	**HP**	**HSP**	**CSP**	**H**	**HS**	**HP**	**HSP**	**CSP**
**4,8-DiMeIQx**	0.03 (0.02)	0.05 (0.02)	0.06 (0.02)	0.20 (0.08)	0.44 (0.05)	*	0.19 (0.07)	0.25 (0.07)	0.75 (0.08)	0.66 (0.11)	0.30 (0.08)	0.17 (0.06)	0.49 (0.08)	0.51 (0.08)	0.85 (0.15)	0.05 (0.02)	0.11 (0.05)	0.15 (0.03)	0.42 (0.08)	0.17 (0.05)
**DMIP**	*	0.11 (0.02)	0.04 (0.02)	0.12 (0.02)	0.06 (0.02)	0.07 (0.02)	0.31 (0.02)	0.09 (0.02)	1.63 (0.14)	1.82 (0.12)	0.05 (0.02)	0.03 (0.02)	0.06 (0.02)	0.44 (0.03)	0.23 (0.02)	0.04 (0.02)	0.05 (0.02)	0.04 (0.02)	0.06 (0.02)	0.59 (0.04)
**IQ**	*	0.20 (0.04)	0.07 (0.04)	0.22 (0.04)	0.08 (0.03)	*	0.26 (0.05)	0.18 (0.05)	0.44 (0.05)	0.70 (0.04)	0.15 (0.04)	0.20 (0.05)	0.18 (0.04)	0.62 (0.05)	0.28 (0.05)	0.04 (0.03)	0.06 (0.04)	0.23 (0.05)	0.82 (0.05)	0.38 (0.04)
**MeIQ**	0.03 (0.02)	0.13 (0.02)	0.07 (0.02)	0.14 (0.02)	0.05 (0.02)	0.07 (0.02)	0.28 (0.02)	0.10 (0.02)	0.54 (0.04)	0.57 (0.04)	0.19 (0.02)	0.44 (0.03)	0.28 (0.02)	0.65 (0.05)	0.46 (0.03)	0.04 (0.02)	0.05 (0.02)	0.29 (0.02)	0.72 (0.05)	0.40 (0.03)
**MeIQx**	*	0.06 (0.01)	*	0.16 (0.02)	*	*	0.07 (0.01)	0.28 (0.03)	0.53 (0.05)	0.37 (0.04)	0.01 (0.01)	0.05 (0.01)	0.11 (0.01)	0.33 (0.03)	0.17 (0.02)	0.15 (0.02)	0.19 (0.02)	0.41 (0.04)	0.82 (0.05)	0.45 (0.04)
**PhIP**	0.03 (0.02)	0.16 (0.02)	0.20 (0.02)	0.31 (0.02)	0.08 (0.02)	*	0.32 (0.04)	0.16 (0.03)	0.93 (0.03)	0.80 (0.03)	0.22 (0.04)	0.11 (0.03)	0.27 (0.03)	0.65 (0.02)	0.32 (0.04)	0.18 (0.03)	0.23 (0.03)	0.21 (0.03)	0.79 (0.02)	0.53 (0.02)
**AαC**	*	0.30 (0.02)	0.17 (0.03)	0.27 (0.02)	0.16 (0.03)	0.03 (0.02)	0.24 (0.02)	0.07 (0.02)	1.19 (0.07)	2.63 (0.05)	0.06 (0.01)	0.10 (0.03)	0.15 (0.02)	0.60 (0.03)	0.19 (0.02)	0.13 (0.01)	0.19 (0.02)	0.55 (0.04)	0.96 (0.10)	0.70 (0.03)
**Me AαC**	*	0.19 (0.07)	0.14 (0.06)	0.22 (0.05)	0.12 (0.05)	*	0.30 (0.08)	0.08 (0.04)	1.11 (0.07)	1.76 (0.05)	*	*	*	0.23 (0.07)	*	0.11 (0.05)	0.17 (0.05)	0.41 (0.10)	0.90 (0.09)	0.46 (0.09)
**Trp-P-1**	*	0.18 (0.06)	0.07 (0.03)	0.23 (0.06)	0.36 (0.07)	0.49 (0.06)	0.22 (0.05)	0.08 (0.03)	1.07 (0.09)	2.72 (0.10)	0.09 (0.03)	0.06 (0.03)	0.22 (0.06)	0.35 (0.04)	0.25 (0.07)	0.24 (0.07)	0.30 (0.05)	0.32 (0.05)	0.83 (0.09)	0.66 (0.08)
**Trp-P-2**	*	0.17 (0.06)	0.13 (0.07)	0.18 (0.03)	0.08 (0.03)	0.04 (0.02)	0.33 (0.04)	0.07 (0.04)	1.17 (0.09)	1.63 (0.07)	*	*	0.04 (0.02)	0.22 (0.07)	0.04 (0.02)	0.06 (0.04)	0.14 (0.05)	0.40 (0.08)	0.85 (0.06)	0.40 (0.09)
**∑ HAAs (µg L^{-1})**	0.09	1.55	0.95	2.05	1.43	0.70	2.52	1.36	9.36	13.66	1.07	1.16	1.80	4.60	2.79	1.04	1.49	3.01	7.17	4.74
**Mean (µg L^{-1})**	0.03	0.16	0.11	0.21	0.16	0.14	0.25	0.14	0.94	1.37	0.13	0.15	0.20	0.46	0.31	0.10	0.15	0.30	0.72	0.47

[a](**H**) Homemade sample, unsweetened and unpasteurized; [b](**HS**) Homemade sample, sweetened and unpasteurized; [c](**HP**) Homemade sample, unsweetened and pasteurized; [d](**HSP**) Homemade sample, sweetened and pasteurized; [e](**CSP**) Commercial sample, sweetened and pasteurized. * < LOD. Each value is reported with its 95% confidence interval (value shown in parentheses).

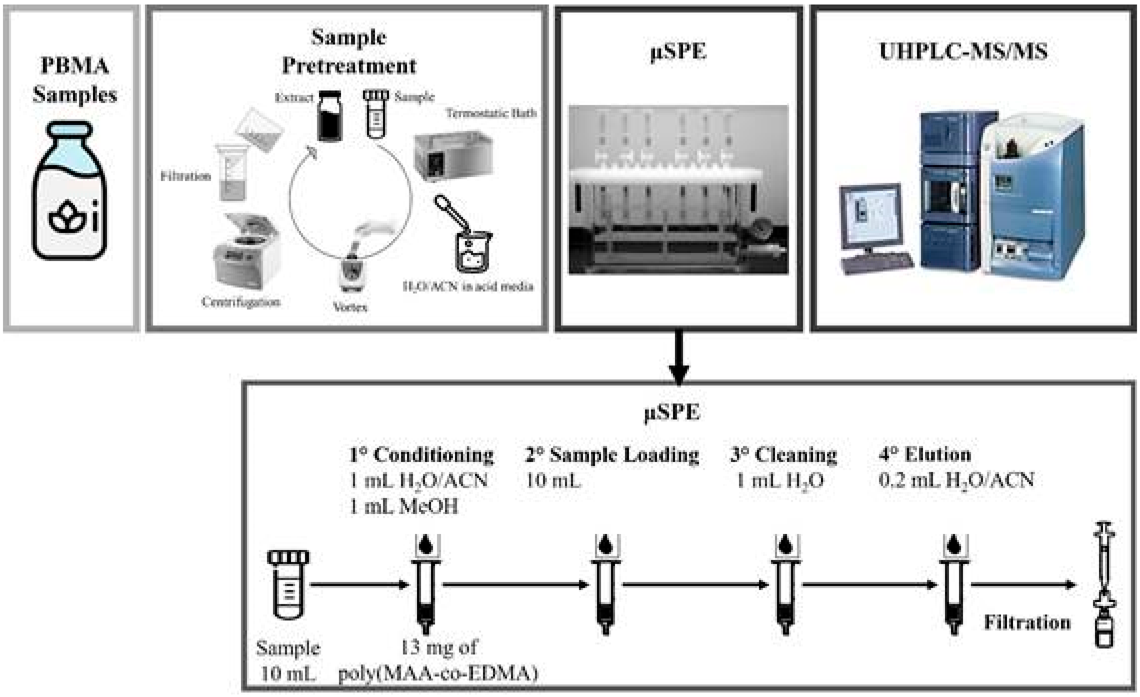

Figure S1. Schematic representation of the solid-phase microextraction procedure followed by high-performance liquid chromatography coupled with tandem mass spectrometry (µSPE-UHPLC-MS/MS).

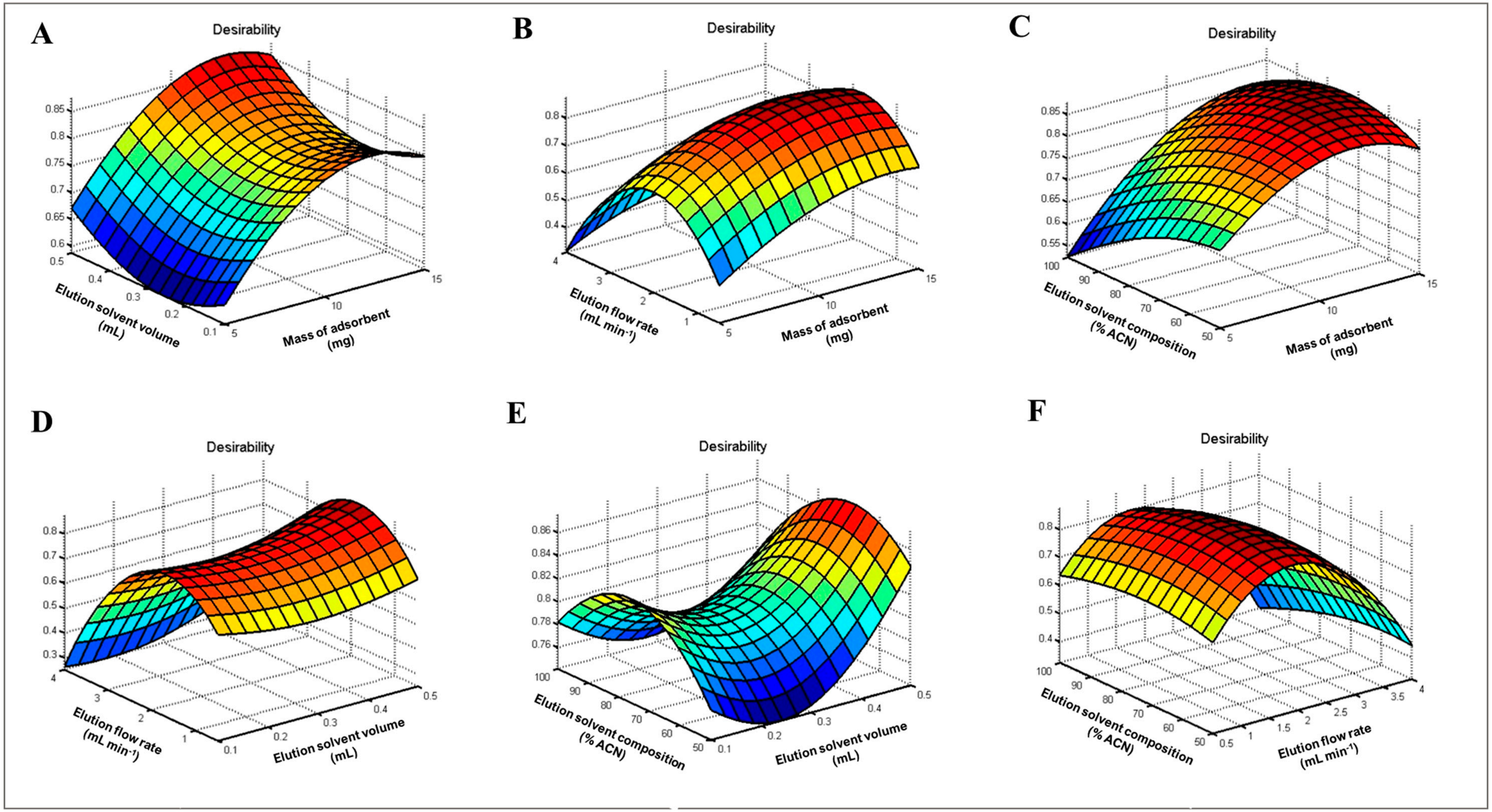

Figure S2. Desirability surfaces obtained by ANN modelling as a function of: **A**. elution solvent volume and mass of adsorbent material; **B**. elution flow rate and mass of adsorbent; **C**. elution solvent composition and mass of adsorbent; **D**. elution flow rate and elution solvent volume; **E**. elution solvent composition and elution solvent volume; and **F**. elution solvent composition and elution flow rate. In each figure, the third factor was maintained at an optimal value.

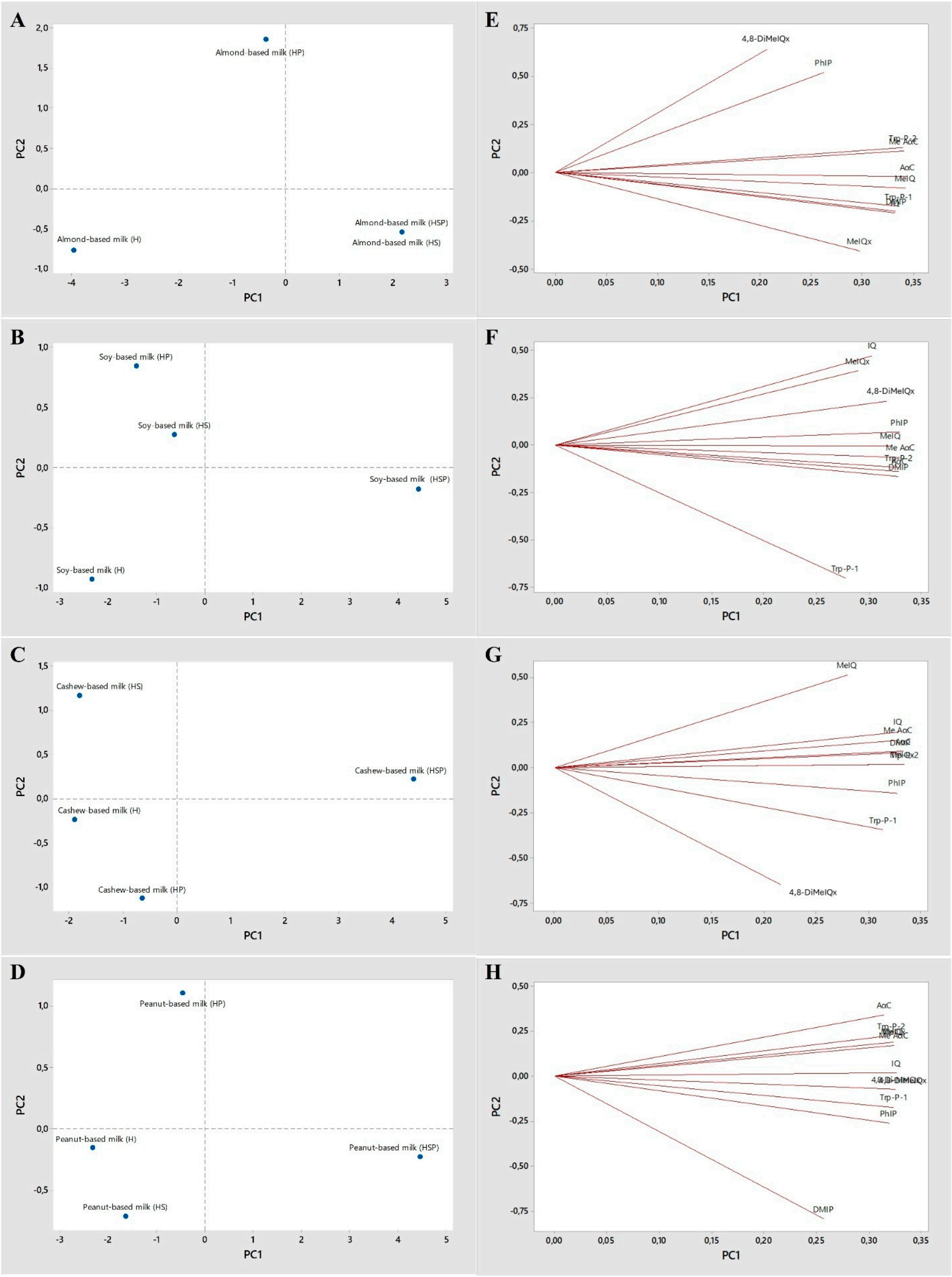

Figure S3. Principal component analysis (PCA) conducted for different PBMAs. Panels **A**, **C**, **E**, and **G** display the PCA scores scatter plots for almond, soy, cashew, and peanut-based milk, respectively. Panels **B**, **D**, **F**, and **H** illustrate the loading plots of PCA for each respective type of milk alternative.

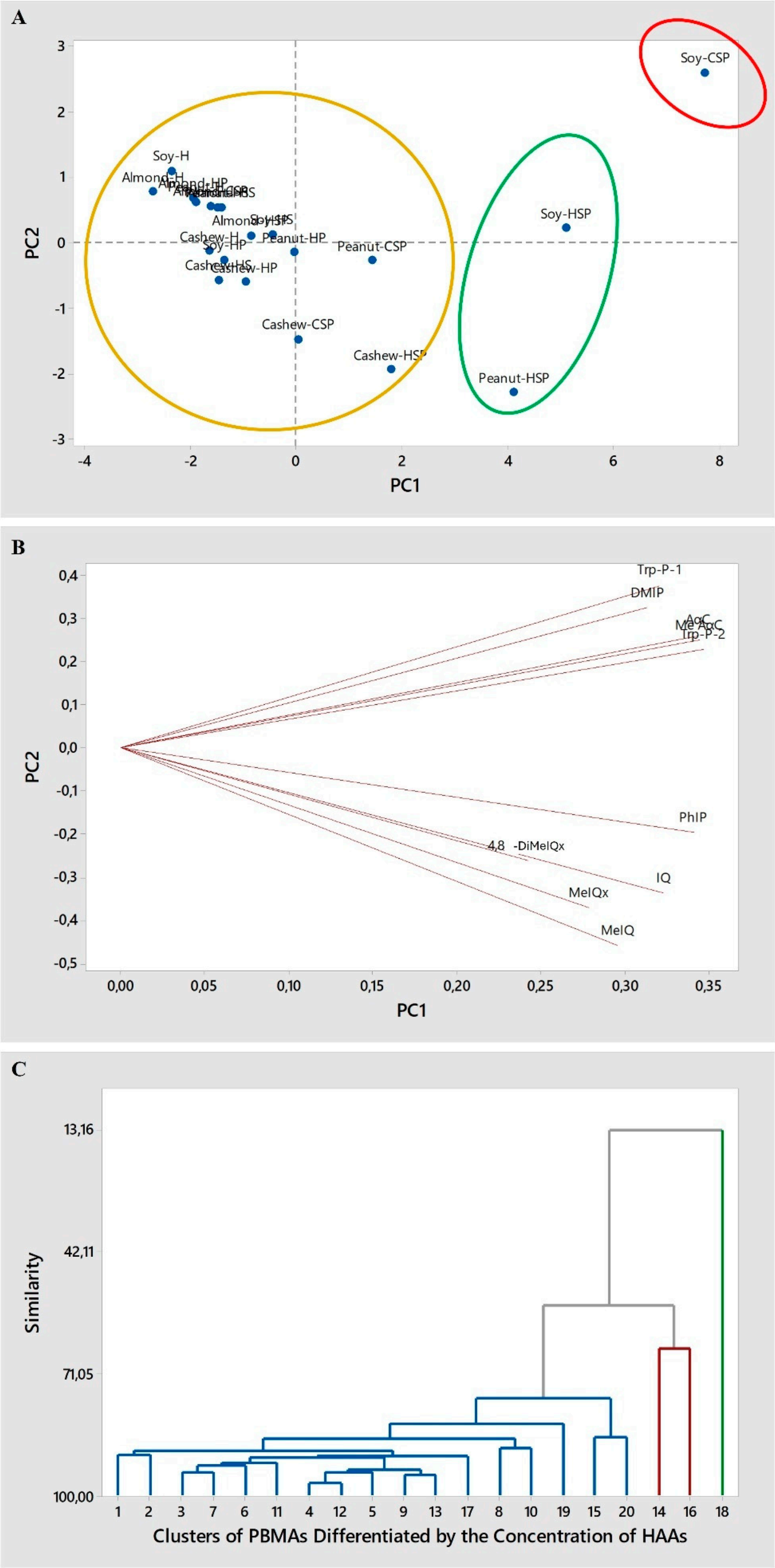

Figure S4. A. Scores plot obtained from principal component analysis for all PBMAs. **B.** Loading plot resulting from the PCA conducted on PBMAs. **C.** Hierarchical clustering dendrogram constructed based on the concentrations of heterocyclic aromatic amines and the classification of PBMAs.
